# Oxidative Stress-Dependent Synergistic Antiproliferation, Apoptosis, and DNA Damage of Ultraviolet-C and Coral-Derived Sinularin Combined Treatment for Oral Cancer Cells

**DOI:** 10.3390/cancers13102450

**Published:** 2021-05-18

**Authors:** Sheng-Yao Peng, Jen-Yang Tang, Ruei-Nian Li, Hurng-Wern Huang, Chang-Yi Wu, Chien-Chih Chiu, Fang-Rong Chang, Hong-Wei Zhang, Yun-Jou Lee, Jyh-Horng Sheu, Hsueh-Wei Chang

**Affiliations:** 1 PhD Program in Life Science, Department of Biomedical Science and Environmental Biology, College of Life Science, Kaohsiung Medical University, Kaohsiung 80708, Taiwan; u109851101@kmu.edu.tw (S.-Y.P.); runili@kmu.edu.tw (R.-N.L.); 2 School of Post-Baccalaureate Medicine, Kaohsiung Medical University, Kaohsiung 80708, Taiwan; reyata@kmu.edu.tw; 3 Department of Radiation Oncology, Kaohsiung Medical University Hospital, Kaohsiung 80708, Taiwan; 4 Institute of Biomedical Science, National Sun Yat-sen University, Kaohsiung 80424, Taiwan; sting@mail.nsysu.edu.tw; 5 Department of Biological Sciences, National Sun Yat-sen University, Kaohsiung 80424, Taiwan; cywu@mail.nsysu.edu.tw; 6 Department of Biotechnology, Kaohsiung Medical University, Kaohsiung 80708, Taiwan; cchiu@kmu.edu.tw; 7 Graduate Institute of Natural Products, Kaohsiung Medical University, Kaohsiung 80708, Taiwan; aaronfrc@kmu.edu.tw (F.-R.C.); u108531007@kmu.edu.tw (Y.-J.L.); 8 Department of Marine Biotechnology and Resources, National Sun Yat-sen University, Kaohsiung 80424, Taiwan; m085020011@student.nsysu.edu.tw; 9 Doctoral Degree Program in Marine Biotechnology, National Sun Yat-sen University, Kaohsiung 80424, Taiwan; 10 Department of Medical Research, China Medical University Hospital, China Medical University, Taichung 40402, Taiwan; 11 Frontier Center for Ocean Science and Technology, National Sun Yat-sen University, Kaohsiung 80424, Taiwan; 12 Institute of Medical Science and Technology, National Sun Yat-sen University, Kaohsiung 80424, Taiwan; 13 Center for Cancer Research, Kaohsiung Medical University, Kaohsiung 80708, Taiwan; 14 Cancer Center, Kaohsiung Medical University Hospital, Kaohsiung 80708, Taiwan

**Keywords:** UVC, sinularin, combined treatment, oral cancer, coral, DNA damage, apoptosis, oxidative stress

## Abstract

**Simple Summary:**

Combined treatments with low side effects enhance anticancer applications. This study focusses on validating the potential synergistic antiproliferation of the combined treatment of ultraviolet-C and the coral-derived compound sinularin (UVC/sinularin) in oral cancer cells. This study confirms that UVC/sinularin synergistically and selectively inhibits oral cancer cell proliferation with low cytotoxicity on normal oral cells. The mechanisms involve the enhanced cellular and mitochondrial oxidative stress that cause apoptosis, DNA damage, and mitochondrial dysfunction in oral cancer cells.

**Abstract:**

Combined treatment is increasingly used to improve cancer therapy. Non-ionizing radiation ultraviolet-C (UVC) and sinularin, a coral *Sinularia flexibilis*-derived cembranolide, were separately reported to provide an antiproliferation function to some kinds of cancer cells. However, an antiproliferation function using the combined treatment of UVC/sinularin has not been investigated as yet. This study aimed to examine the combined antiproliferation function and explore the combination of UVC/sinularin in oral cancer cells compared to normal oral cells. Regarding cell viability, UVC/sinularin displays the synergistic and selective killing of two oral cancer cell lines, but remains non-effective for normal oral cell lines compared to treatments in terms of MTS and ATP assays. In tests using the flow cytometry, luminescence, and Western blotting methods, UVC/sinularin-treated oral cancer cells exhibited higher reactive oxygen species production, mitochondrial superoxide generation, mitochondrial membrane potential destruction, annexin V, pan-caspase, caspase 3/7, and cleaved-poly (ADP-ribose) polymerase expressions than that in normal oral cells. Accordingly, oxidative stress and apoptosis are highly induced in a combined UVC/sinularin treatment. Moreover, UVC/sinularin treatment provides higher G2/M arrest and γH2AX/8-hydroxyl-2′deoxyguanosine-detected DNA damages in oral cancer cells than in the separate treatments. A pretreatment can revert all of these changes of UVC/sinularin treatment with the antioxidant *N*-acetylcysteine. Taken together, UVC/sinularin acting upon oral cancer cells exhibits a synergistic and selective antiproliferation ability involving oxidative stress-dependent apoptosis and cellular DNA damage with low toxic side effects on normal oral cells.

## 1. Introduction

Radiation and chemotherapy are the routine remedies for oral cancer therapy [1]. The problems of radio- and chemo-resistance, and their associated side effects, may weaken their anticancer applications in oral cancer therapies [2,3,4]. Combined treatment with natural products provides an improved strategy to overcome radio- and chemo-resistance or side effects in cancer therapy [5,6]. For the complex etiology and targeting of cancer, combinations of anticancer drugs, natural products and radiation are increasingly used to improve the therapeutic efficiency for oral cancer [7,8].

Although X-rays are commonly used in radiotherapy, anticancer studies using nonionizing radiation are increasing. For example, ultraviolet-C (UVC) exhibited an antiproliferation effect to pancreatic [9] and colon [10] cancer cells. A UVC-generator is cheaper and more portable than an X-ray machine [11]. UVC irradiation may be effortlessly operated in the clinic, rather than the medical center. UVC exposure shows translational potential to inhibit the proliferation of glioblastoma multiforme cancer cells [12]. The combined treatments of clinical drugs [13,14], chemical agents [15], or natural products [16,17] with UVC irradiation may improve the antiproliferation of several types of cancer cells. Moreover, UVC irradiation inhibits tumor growth in an animal model without apparent side effects [11,18]. Therefore, the discovery of the drug amendments applied as UVC radiosensitizers may effectively improve oral cancer therapy.

Sinularin is one of the main natural products in soft corals, such as *Sinularia flexibilis* [19] and *S. manaarensis* [20]. The antiproliferation property of sinularin was demonstrated in a panel of cancer cells such as melanoma (A2058) [21], gastric (AGS) [22], liver (HepG2) [23], oral (Ca9-22) [24], breast (SKBR3) [25], and renal (786-O) [26] cell lines. However, these studies did not examine the drug safety of sinularin in non-malignant cell types.

The selective killing effect of sinularin was firstly reported in our previous works on oral [24] and breast [25] cancer cells, and it was subsequently mentioned in renal cancer cells [26]. These studies reported that sinularin showed low cytotoxicity to non-malignant cells of oral (HGF-1), breast (M10), and renal (HRCEpic) origins [24,25,26], suggesting that sinularin shows low side effects on normal cells.

Currently, the effects of a combined treatment using UVC and sinularin (UVC/sinularin) in oral cancer therapy have not been reported. We hypothesized that UVC/sinularin exhibited the synergistic and selective killing of oral cancer cells. In order to validate the hypothesis, the survival, cell cycle distribution, apoptotic change, cellular oxidative stress imbalance, and induced DNA damage following the separate and combined treatments (UVC and/or sinularin) were compared between cancer and normal types of oral cells. For the above endpoints, we employed ATP analysis, flow cytometry, and Western blotting. The therapeutic goal of this study is to examine the potential anticancer application for UVC/sinularin combined treatment in the example of gingival and tongue cancer cells.

## 2. Materials and Methods

### 2.1. Cell Lines

The human gingival and tongue cancer (Ca9-22 and CAL 27) cell lines and normal (HGF-1) oral cell lines were from JCRB Cell Bank and ATCC, located in Osaka, Japan, and Manassas, VA, USA, respectively. The cell culture materials were purchased from Gibco (Grand Island, NY, USA). All of the cells were cultured with a medium supplemented with 10% fetal bovine serum and antibiotics in an incubator with 5% CO_2_ and humidified 37 °C atmospheres. The media for the oral cancer and normal oral cells were 3:2 and 4:1 mixtures of Dulbecco’s Modified Eagle Medium (DMEM) and F12 [27].

### 2.2. UVC Irradiation, Sinularin Preparation, Chemicals and Cell Treatments

Following the removal of the culture medium, the human oral cells were placed in a laminar flow and irradiated with the built-in UVC (254 nm) lamp at the dose rate of 2 J/m^2^/sec [16] for the required periods, i.e., 5 sec for 10 J/m^2^ (for CAL 27 cells) and 6 s for 12 J/m^2^ (for Ca9-22 and HGF-1 cells). The non-UVC irradiated control cells followed the same procedure except for the UVC irradiation.

The sinularin was prepared by the standard purification processes using cultured soft coral *S. manaarensis,* as previously mentioned [20], and dissolved in DMSO. The ^1^H-NMR, ^13^C-NMR spectra and the HPLC profile are provided in Figures S1–S3 as evidence for the purity of the sinularin.

*N*-acetylcysteine (NAC), the reactive oxygen species (ROS) inhibitor (Sigma-Aldrich, St. Louis, MO, USA) [28,29,30,31,32], was chosen for preincubation (5 mM, 1 h) before the UVC and/or sinularin treatments in order to confirm the involvement of oxidative stress. Z-VAD-FMK (ZVAD), the pancaspase inhibitor (Selleckchem.com; Houston, TX, USA), was chosen for preincubation (100 μM for 2 h) before UVC and/or sinularin treatments to confirm the involvement of apoptosis. All of the reagents contained the same concentration of 0.1% DMSO.

Following the NAC or ZVAD pretreatments or not, the cells were arranged into four kinds of treatments: the control (0.1% DMSO in medium), UVC, sinularin, and UVC/sinularin for 48 h. The UVC and/or sinularin conditions were 12 J/m^2^, 2 μM and 10 J/m^2^, 3 μM for oral cancer cells (Ca9-22, CAL 27), and 12 J/m^2^, 3 μM for normal cells (HGF-1) for 48 h, respectively.

### 2.3. Survival Analyses by MTS and ATP Assays

The cell survival was determined by both MTS (Promega Corporation, Madison, WI, USA) [33] and ATP assays (PerkinElmer Life Sciences, Boston, MA, USA) [34]. Briefly, the MTS substrate was mixed with the medium to a final concentration of 58.1 µg/mL at 37 °C for 1 h in the dark for the MTS assay. Subsequently, it was detected by the Epoch™ Microplate Spectrophotometer (Bio Tek; Winooski, USA). For the ATP assay, 100 μL of the cell lysate was mixed with 12.5 μL of the reaction substrate (D-luciferin and luciferase) at room temperature for 5 min in the dark. Subsequently, it was detected by a CentroLIApc LB 962 Microplate Luminometer (Berthold Technologies GmbH & Co., Bad Wildbad, Germany). The synergy between the UVC/sinularin was calculated as previously described [35], i.e., the synergy (α) = the survival fraction for the single treatment (UVC) × the survival fraction for the single treatment (sinularin)/the survival fraction for the combined treatment (UVC/sinularin). The additive, synergistic, or antagonistic effects results were: α = 1, > 1, and < 1, respectively.

### 2.4. Cell Cycle Detection

For the cell cycle assay, Ca9-22 and CAL 27 cells were harvested with trypsin, fixed with 70% ethanol, washed with PBS, and stained with Biotium DNA dye (Hayward, CA, USA), i.e., 7-aminoactinmycin D (7AAD) at the final concentration of 10 μg/mL (37 °C, 30 min) [36]. Subsequently, the cell cycle phase distribution for the stained cells was measured using a Guava easyCyte flow cytometer (Luminex, Austin, TX, USA). The data were analyzed by Becton-Dickinson FlowJo (Franklin Lakes, NJ, USA).

### 2.5. Apoptotic Annexin V Flow Cytometry and Western Blotting

The apoptosis was determined by two kinds of flow cytometry analyses, i.e., annexin V (Strong Biotech Corporation, Taipei, Taiwan)/7AAD [37] and pan-caspase activity detection (Abcam, Cambridge, UK) [34]. Briefly, the cells (Ca9-22, CAL 27, and HGF-1) were either incubated in the binding buffers containing the annexin V-FITC (1:1000 dilution)/7AAD (1 μg/mL) or the pancaspase common substrate TF2-VAD-FMK (1:100,000 dilution) at 37 °C for 30 min.

For the apoptosis signaling, the apoptosis-related activities were examined by Caspase-Glo^®^ 3/7 commercial product (Promega; Madison, WI, USA) [15] and Western blotting. Briefly, a luminogenic caspase-3/7 tetrapeptide substrate (DEVD) and buffer were equally mixed and incubated at 37 °C for 30 min in the dark. Subsequently, this mixture was applied to a 96-well plate for the measuring of the caspase 3/7 activity of the drug-treated cells. For the Western blotting, Cell Signalling Technology cleaved poly (ADP-ribose) polymerase (c-PARP) antibody (Danvers, MA, USA) (1:1000 dilution) was used to detect apoptosis, and Sigma-Aldrich β-actin was included for the detection of the loading control [38]. Figure S4: Original western blotting figures.

### 2.6. ROS Flow Cytometry

The ROS specific dye 2′,7′-dichlorodihydrofluorescein diacetate was obtained from Sigma-Aldrich. Following the cell harvesting and PBS washing, the cell treatment condition for this ROS dye was applied at 10 μM (37 °C, 30 min) in the dark, was reacted in proportion to cellular ROS amount, and became a fluorescence chemical reaction for the flow cytometry and FlowJo analysis [28]. The cells (Ca9-22, CAL 27, and HGF-1) were resuspended in PBS before the flow cytometry.

### 2.7. Mitochondrial Superoxide (MitoSOX) Flow Cytometry

Following the cell harvesting and PBS washing, the cells were incubated with MitoSOX Red (Invitrogen, Eugene, OR, USA) (50 nM, 37 °C, 30 min) in the dark. MitoSOX can react in proportion to the MitoSOX amount, and can generate fluorescence for flow cytometry and FlowJo analysis [25]. The cells (Ca9-22, CAL 27, and HGF-1) were resuspended in PBS before the flow cytometry.

### 2.8. Mitochondrial Membrane Potential (MitoMP) Flow Cytometry

Following the cell harvesting and PBS washing, the cells were incubated with DiOC_2_(3) (Invitrogen) (5 nM, 37 °C, 30 min) in the dark. DiOC_2_(3) can primarily accumulate in mitochondria in proportion to MitoMP, and it generates the fluorescence detected by flow cytometry and FlowJo analysis [39]. The cells (Ca9-22, CAL 27, and HGF-1) were resuspended in PBS before the flow cytometry.

### 2.9. Quantitative RT-PCR (qRT-PCR) Assay for the mRNA Expression of Antioxidant Signaling Genes

The RNA and cDNA preparations were performed [40] for qRT-PCR using a touch-down PCR program, as previously described [41]. The primer sequences (5′-> 3′) [42,43,44] for the antioxidant signaling genes [42,45,46] were described as follows: Nuclear factor erythroid 2-like 2 (*NFE2L2*; *NRF2*)-Forward (F): GATCTGCCAACTACTCCCAGGTT and *NFE2L2*-Reverse (R): CTGTAACTCAGGAATGGATAATAGCTCC, superoxide dismutase 1 (*SOD1*)-F: AGGGCATCATCAATTTCGAGC and *SOD1*-R: CCCAAGTCTCCAACATGCCTC, thioredoxin (*TXN*)-F: GAAGCAGATCGAGAGCAAGACTG and *TXN*-R: GCTCCAGAAAATTCACCCACCT, glutathione-disulfide reductase (*GSR*)-F: GTTCTCCCAGGTCAAGGAGGTTAA and *GSR*-R: CCAGCAGCTATTGCAACTGGAGT, catalase (*CAT*)-F: ATGCAGGACAATCAGGGTGGT and *CAT*-R: CCTCAGTGAAGTTCTTGACCGCT, glutathione peroxidase 1 (*GPX1*)-F: AACCAGTTTGGGCATCAGGAG and *GPX1*-R: AGTTCCAGGCAACATCGTTGC, were analyzed. In the reference of the housekeeping gene *GAPDH* [43,44] (F: CCTCAACTACATGGTTTACATGTTCC and R: CAAATGAGCCCCAGCCTTCT), their relative mRNA expressions (log_2_) were measured by the 2^−ΔΔCt^ method [47].

### 2.10. γH2AX Flow Cytometry

Following the cell harvesting and PBS washing, the DNA double-strand break damage was determined by antibody-associated flow cytometry [48]. Briefly, the cells (Ca9-22 and CAL 27) were fixed and subsequently processed with Santa Cruz Biotechnology antibody for γH2AX (Santa Cruz, CA, USA) (1:500 dilution). Its matched Cell Signaling Technology antibody was marked with Alexa Fluor 488 (1:10,000 dilution) and 7AAD staining (5 μg/mL) for the flow cytometry and FlowJo analysis. The cells were resuspended in PBS before the flow cytometry.

### 2.11. 8-Hydroxyl-2′-Deoxyguanosine (8-OHdG) Flow Cytometry

Following the cell harvesting and PBS washing, the oxidative DNA damage (8-OHdG) was determined by antibody-associated flow cytometry [35]. Generally, the fixed cells (Ca9-22 and CAL 27) were mixed with 8-OHdG-FITC antibody (1:10,000 dilution) (Santa Cruz Biotechnology) (4 °C, 1 h) for the flow cytometry and FlowJo analysis. The cells were resuspended in PBS before the flow cytometry.

### 2.12. Statistics

**Figure 1:**
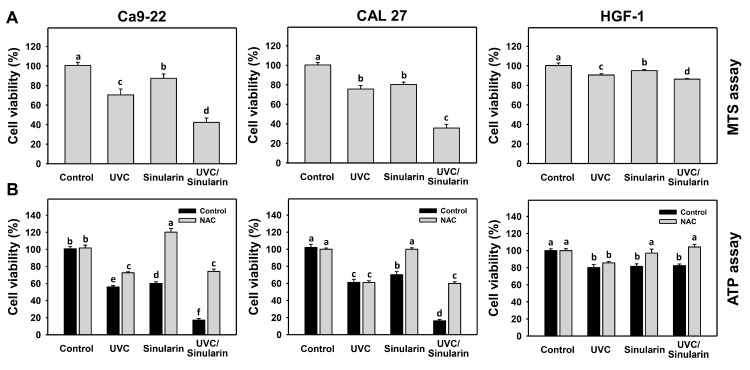
Cell viability for UVC and/or sinularin using MTS and ATP detections. Following the NAC preincubation (5 mM for 1 h) or not, the cells were arranged into four kinds of treatments: the control (0.1% DMSO in medium), UVC, sinularin, and UVC/sinularin for 48 h. UVC and/or sinularin conditions were 12 J/m^2^, 2 μM and 10 J/m^2^, 3 μM for oral cancer cells (Ca9-22, CAL 27), and 12 J/m^2^, 3 μM for normal cells (HGF-1) for 48 h, respectively. Subsequently, the cell viability was detected by MTS and ATP assays. (**A**) MTS assay. (**B**) ATP assay. For multi-comparisons, the treatments marked without repeated characters (a to f) differ significantly (*p* < 0.05). In the example of Ca9-22 cells (Figure 1B), the viabilities between the control (black) and NAC (gray) for the treatments of UVC (e vs. c), sinularin (d vs. a), and UVC/sinularin (f vs. c) showing different characters indicate significant differences. In comparison, the viabilities between the control and NAC for the control treatments, showing the same character (b), indicate non-significant differences. Similarly, the control (black) viabilities for the treatments of UVC (e vs. b), sinularin (d vs. b), and UVC/sinularin (f vs. b) showing different characters indicate significant differences. The data were plotted as the mean ± SD (*n* = 3).

Except for the Western blotting results (Student’s *t*-test), the one-way ANOVA evaluation and Tukey HSD post hoc examination (JMP software) were used for the statistical analysis for multi-comparisons. The treatments marked without overlapping characters differ significantly. The example to illustrate the meaning of the significance is shown at the end of the figure legend in Figure 1.

## 3. Results

### 3.1. UVC and Sinularin (UVC/Sinularin) Combined Treatment of Oral Cancer Cells Shows Synergistic and Selective Killing

For the 48 h MTS assay (Figure 1A), UVC/sinularin-treated oral cancer cells showed lower viability for 42.3% and 35.8% than UVC (12 or 10 J/m^2^) or sinularin (2 or 3 μM) alone in oral cancer Ca9-22 (70.4% or 87.4%) and CAL 27 (75.7% or 80.3%) cells. However, UVC and/or sinularin only showed low cytotoxicity (around 90% viability) in normal oral HGF-1 cells.

For the 48 h ATP assay (Figure 1B), UVC/sinularin-treated oral cancer cells showed lower viability for 16.1% and 15.9% than UVC or sinularin alone in oral cancer Ca9-22 (56.0% or 60.1%) and CAL 27 (60.7% or 68.7%) cells. However, UVC and/or sinularin for normal HGF-1 cells only showed low cytotoxicity (around 80% viability). Moreover, the oxidative stress involvement in modulating the cell viability was estimated by the presence of antioxidant NAC. NAC rescues the antiproliferation of sinularin and UVC/sinularin treatments in both oral cancer cell lines, although NAC shows different responses to UVC between oral cancer cells. NAC also rescues the mild antiproliferation to normal oral cells.

The synergy determinations (α values) of UVC/sinularin in oral cancer cells for the MTS assay (Ca9-22 vs. CAL 27) are 1.46 ± 0.05 and 1.71 ± 0.11 (Figure 1A), and for the ATP assay are 2.09 ± 0.11 and 2.62 ± 0.08 (Figure 1B), respectively. This demonstrates that UVC/sinularin combined treatment shows a synergistic antiproliferation effect on oral cancer cells.

### 3.2. UVC/Sinularin Combined Treatment of Oral Cancer Cells Highly Induces Cell Cycle Disturbance

**Figure 2:**
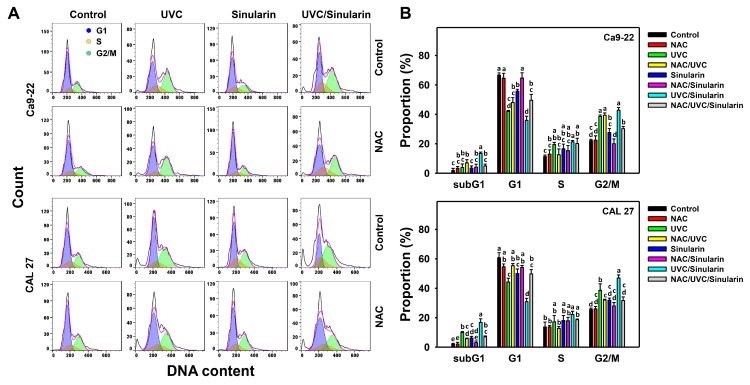
Cell cycle analysis for the UVC and/or sinularin treatments. Following the NAC preincubation (5 mM for 1 h) or not, the cells were arranged into four kinds of treatments: the control (0.1% DMSO in medium), UVC, sinularin, and UVC/sinularin for 48 h. The UVC and/or sinularin conditions were 12 J/m^2^, 2 μM and 10 J/m^2^, 3 μM for oral cancer cells (Ca9-22, CAL 27) for 48 h, respectively. (**A**,**B**) Patterns and quantifications for the cell cycle analysis. For multi-comparisons of the same cell cycle phase, the treatments marked without repeated characters (a to e) differ significantly (*p* < 0.05). The data were plotted as the means ± SD (*n* = 3).

The oral cell cycle progression pattern following the four types of treatments—the control, UVC, sinularin, and UVC/sinularin—is displayed for Ca9-22 and CAL 27 cells (Figure 2A). For these oral cancer cells, the UVC/sinularin treatment exhibited more sub-G1 (%) and G2/M (%), and it shows lower G1 (%) than the separate treatments (UVC or sinularin) as well as the control (Figure 2B).

Moreover, the involvement of an oxidative stress-modulating effect on the cell cycle disturbance on Ca9-22 and CAL 27 cells was estimated by the presence of antioxidant NAC (Figure 2A). NAC suppresses the subG1 and G2/M inductions and G1 reduction to the UVC/sinularin treatments in both oral cancer cell lines (Figure 2B).

### 3.3. UVC/Sinularin Combined Treatment of Oral Cancer Cells Highly Induces Annexin V-, Caspase- and Western Blotting-Detected Apoptosis

**Figure 3:**
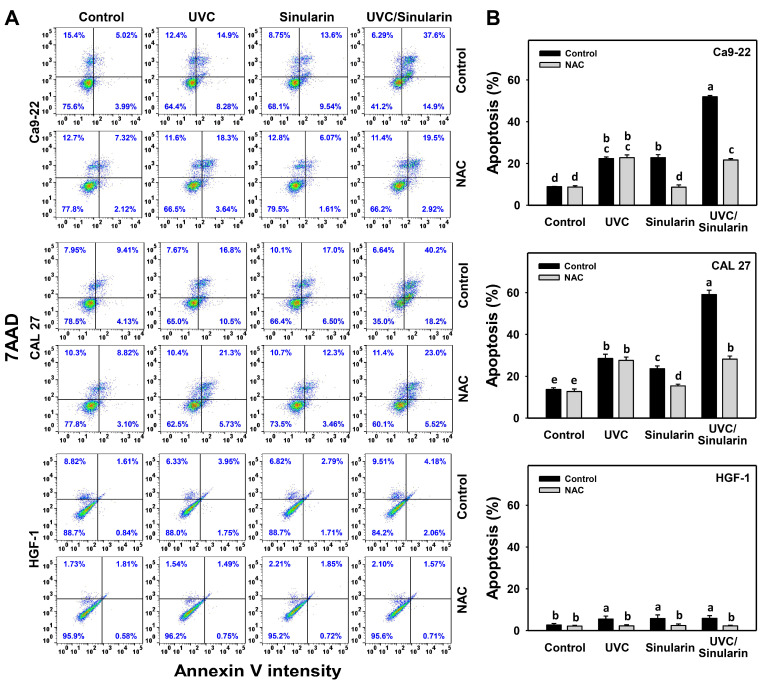
Annexin V-detected apoptosis assays of UVC and/or sinularin treatments. Following the NAC preincubation (5 mM for 1 h) or not, the cells were arranged into four kinds of treatments: the control (0.1% DMSO in medium), UVC, sinularin, and UVC/sinularin for 48 h. The UVC and/or sinularin conditions were 12 J/m^2^, 2 μM and 10 J/m^2^, 3 μM for oral cancer cells (Ca9-22, CAL 27), and 12 J/m^2^, 3 μM for normal cells (HGF-1) for 48 h, respectively. (**A**,**B**) Patterns and quantifications for annexin V/7AAD analysis. The apoptosis (%) is annexin V-positive (%). For multi-comparisons, the treatments marked without repeated characters (a to e) differ significantly (*p* < 0.05). The data were plotted as the mean ± SD (*n* = 3).

The possibility of apoptosis for the UVC/sinularin treatments in Ca9-22 and CAL 27 cells showing higher subG1 (Figure 2) than others was further tested by annexin V/7AAD, pan-caspase, Cas 3/7, and Western blotting analyses, as follows. In the annexin V/7AAD assay, after 48 h of drug treatment (Figure 3A), UVC/sinularin in oral cancer Ca9-22 and CAL 27 cells demonstrated higher annexin V (+) (%) than the separate treatments and control (Figure 3B). However, the UVC and/or sinularin treatments displayed only mild annexin V (+) (%) to normal HGF-1 cells.

Moreover, the oxidative stress involvement in modulating the apoptosis in terms of annexin V expression was estimated by the presence of NAC (Figure 3). NAC rescues the annexin V-detected apoptosis of sinularin and UVC/sinularin treatments in both oral cancer cell lines. NAC also rescues the mild annexin V-detected apoptosis to normal oral cells.

**Figure 4:**
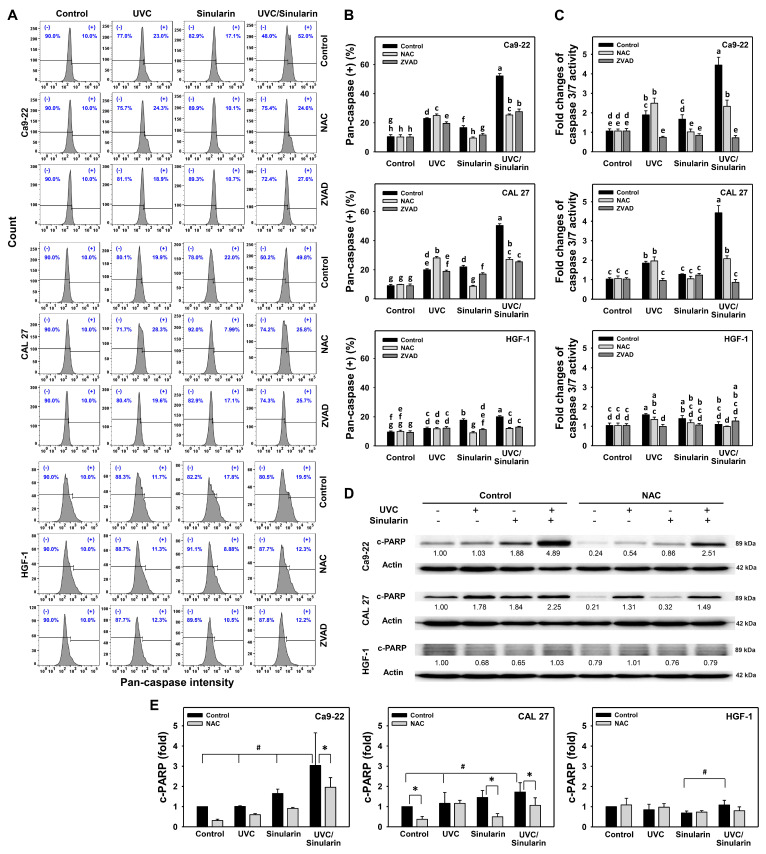
Pan-caspase flow cytometry, Cas 3/7 luminescence assay, and apoptosis Western blotting of the UVC and/or sinularin treatments. Following the NAC (5 mM for 1 h) or ZVAD preincubations (100 μM for 2 h) or not, the cells were arranged into four kinds of treatments: the control (0.1% DMSO in medium), UVC, sinularin, and UVC/sinularin for 48 h. The UVC and/or sinularin conditions were 12 J/m^2^, 2 μM and 10 J/m^2^, 3 μM for oral cancer cells (Ca9-22, CAL 27), and 12 J/m^2^, 3 μM for normal cells (HGF-1) for 48 h, respectively. (**A**,**B**) Pattern and quantifications for the pan-caspase analysis: (+) indicates pan-caspase-positive (%). (**C**) Luminescent assay for caspase 3/7 activity. For multi-comparisons, the treatments marked without repeated characters (a to h) differ significantly (*p* < 0.05). The data were plotted as the mean ± SD (*n* = 3). (**D**,**E**) c-PARP expression and statistical analysis for apoptosis detection. The Student *t*-test analyzed the significance. # was compared between UVC/sinularin and other treatments in the absence of NAC (*p* < 0.05). * was the comparison between the pair treatment in the presence and absence of NAC (*p* < 0.05).

Those annexin V-detected apoptosis expressions were further evaluated by pan-caspase (Figure 4A,B) and Cas 3/7 assays (Figure 4C). For the pan-caspase assay (Figure 4A), UVC/sinularin triggers higher pan-caspase (+) (%), i.e., about 50%, than UVC, sinularin, and the control in two oral cancer cell lines (Ca9-22 and CAL 27) (Figure 4B). For normal oral HGF-1 cells, UVC and/or sinularin trigger low levels of pan-caspase (+) (%), i.e., around 10 to 20%.

For the Cas 3/7 assay, UVC/sinularin for oral cancer Ca9-22 and CAL 27 cells also activated more Cas 3/7 activity (~4 folds) than UVC, sinularin, and the control. Still, the Cas 3/7 activity for the separate and combined treatments was similar in normal HGF-1 cells (1 fold) (Figure 4C). In order to further validate the roles of oxidative stress and apoptosis, their acting inhibitors, such as NAC and ZVAD, were pretreated. For oral cancer cells, both NAC and ZVAD preincubations suppressed the pan-caspase activity for UVC/sinularin to a similar degree, while ZVAD suppressed more Cas 3/7 activity for UVC/sinularin than NAC preincubation.

For the Western blotting (Figure 4D,E), UVC/sinularin overexpressed the apoptotic c-PARP protein in oral cancer Ca9-22 and CAL 27 cells than UVC, sinularin, and the control, but showed a weak expression for c-PARP in normal oral cells. These UVC/sinularin-induced c-PARP expressions in oral cancer cells are inhibited by NAC preincubation.

### 3.4. UVC/Sinularin Combined Treatment of Oral Cancer Cells Highly Induces ROS Production

**Figure 5:**
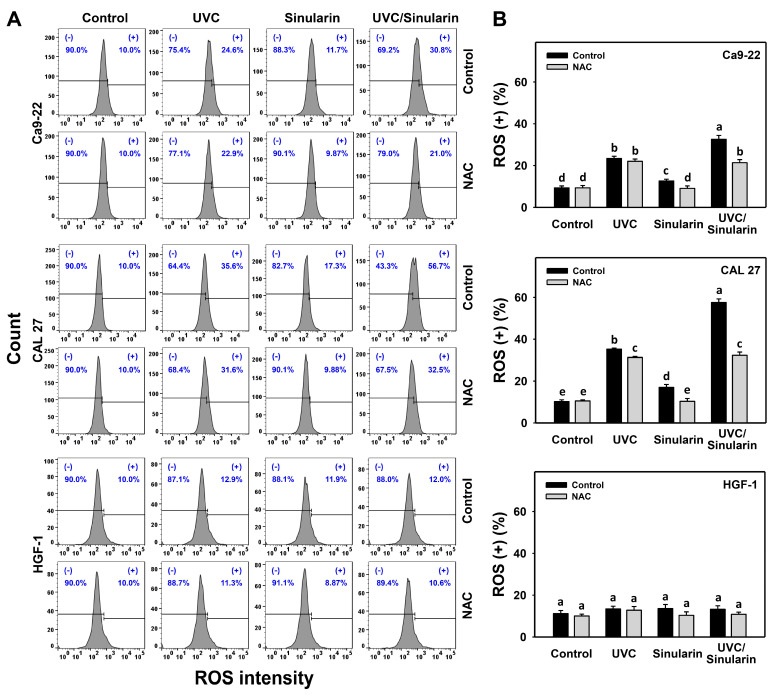
ROS expressions of UVC and/or sinularin treatments. Following the NAC preincubation (5 mM for 1 h) or not, the cells were arranged into four kinds of treatments: the control (0.1% DMSO in medium), UVC, sinularin, and UVC/sinularin for 24 h. The UVC and/or sinularin conditions were 12 J/m^2^, 2 μM and 10 J/m^2^, 3 μM for oral cancer cells (Ca9-22, CAL 27), and 12 J/m^2^, 3 μM for normal cells (HGF-1) for 24 h, respectively. (**A**,**B**) Patterns and quantifications for ROS analysis. (+) indicates ROS-positive (%). For the multi-comparisons, the treatments marked without repeated characters (a to e) differ significantly (*p* < 0.05). The data were plotted as the mean ± SD (*n* = 3).

In the ROS assay (Figure 5A), UVC/sinularin exhibited higher ROS (+) (%) than UVC, sinularin, and the control in two oral cancer cell lines (Ca9-22 and CAL 27) (Figure 5B). However, the ROS changes following UVC and/or sinularin for normal HGF-1 cells maintained basal levels. Moreover, the oxidative stress involvement to modulate the ROS generation was estimated by the presence of NAC (Figure 5). NAC reverted the ROS induction of sinularin and UVC/sinularin treatments in both oral cancer cell lines.

### 3.5. UVC/Sinularin Combined Treatment of Oral Cancer Cells Highly Induces MitoSOX Generation

**Figure 6:**
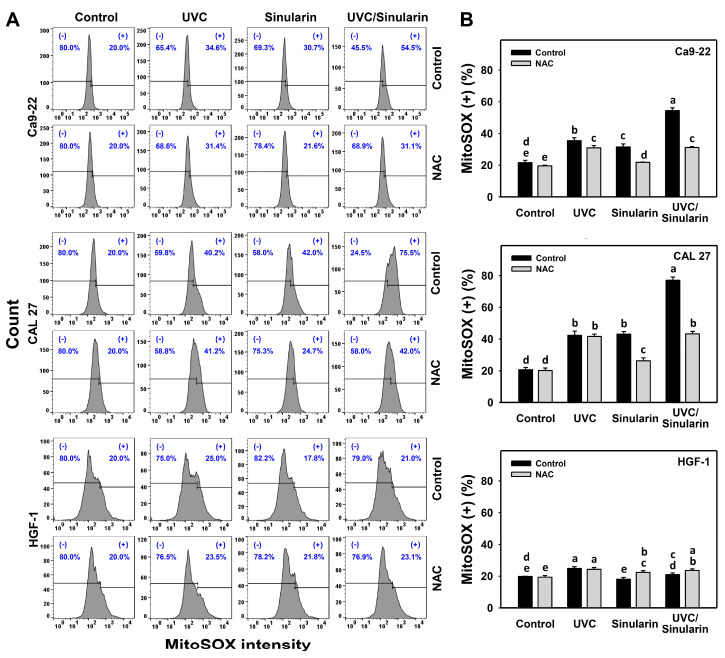
MitoSOX expressions of UVC and/or sinularin treatments. Following the NAC preincubation (5 mM for 1 h) or not, the cells were arranged into four kinds of treatments: the control (0.1% DMSO in medium), UVC, sinularin, and UVC/sinularin for 48 h. The UVC and/or sinularin conditions were 12 J/m^2^, 2 μM and 10 J/m^2^, 3 μM for oral cancer cells (Ca9-22, CAL 27), and 12 J/m^2^, 3 μM for normal cells (HGF-1) for 48 h, respectively. (**A**,**B**) Patterns and quantifications for the MitoSOX analysis. (+) indicates MitoSOX-positive (%). For the multi-comparisons, the treatments marked without repeated characters (a to e) differ significantly (*p* < 0.05). The data were plotted as the mean ± SD (*n* = 3).

In the MitoSOX assay (Figure 6A), UVC/sinularin exhibited higher MitoSOX (+) (%) than UVC, sinularin and the control in two oral cancer cell lines (Ca9-22 and CAL 27) (Figure 6B). However, the MitoSOX changes following UVC and/or sinularin for normal oral HGF-1 cells maintained basal levels. Moreover, the oxidative stress involvement to modulate the MitoSOX change was estimated by the presence of NAC (Figure 6). NAC reverted the MitoSOX induction of sinularin and UVC/sinularin treatments in both oral cancer cell lines.

### 3.6. Oral Cancer Cells Following UVC/Sinularin Combined Treatment Highly Induce MitoMP Destruction

**Figure 7:**
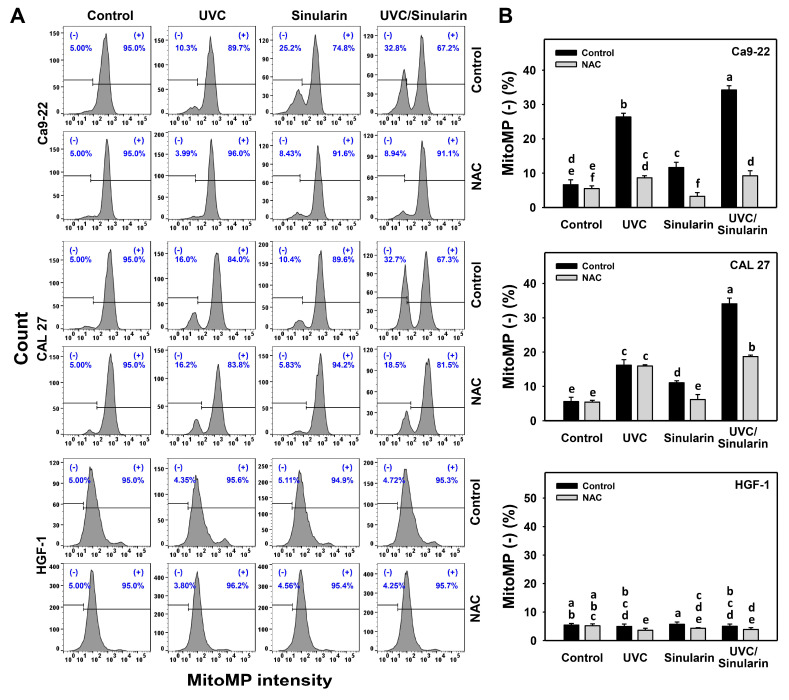
MitoMP expressions of UVC and/or sinularin treatments. Following the NAC preincubation (5 mM for 1 h) or not, the cells were arranged into four kinds of treatments: the control (0.1% DMSO in medium), UVC, sinularin, and UVC/sinularin for 48 h. The UVC and/or sinularin conditions were 12 J/m^2^, 2 μM and 10 J/m^2^, 3 μM for oral cancer cells (Ca9-22, CAL 27), and 12 J/m^2^, 3 μM for normal cells (HGF-1) for 48 h, respectively. (**A**,**B**) Patterns and quantifications for MitoMP analysis. (-) indicates MitoMP-negative (%). For the multi-comparisons, the treatments marked without repeated characters (a to f) differ significantly (*p* < 0.05). The data were plotted as the mean ± SD (*n* = 3).

In the MitoMP assay (Figure 7A), UVC/sinularin exhibited higher MitoMP (-) (%) than UVC, sinularin, and the control in two oral cancer cell lines (Ca9-22 and CAL 27) (Figure 7B). However, the MitoMP changes following UVC and/or sinularin for normal HGF-1 cells maintained basal levels. Moreover, the oxidative stress involvement to modulate the MitoMP change was estimated by the presence of NAC (Figure 7). NAC reverted the MitoMP destruction of sinularin and UVC/sinularin treatments in both oral cancer cell lines.

### 3.7. UVC/Sinularin Combined Treatment of Oral Cancer Cells Downregulates Antioxidant Gene Expressions

**Figure 8:**
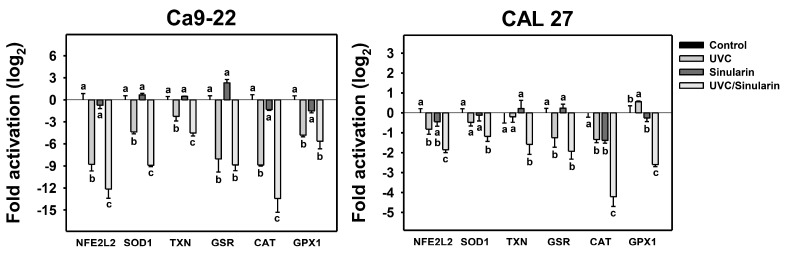
Antioxidant gene expression assay of the UVC and/or sinularin treatments. The fold activation (log_2_) of a panel of antioxidant genes (*NFE2L2*, *SOD1*, *TXN*, *GSR*, *CAT*, and *GPX1*) was performed by qPCR. The UVC and/or sinularin conditions were 12 J/m^2^, 2 μM and 10 J/m^2^, 3 μM for oral cancer cells (Ca9-22, CAL 27) for 48 h, respectively. For the multi-comparisons, the treatments marked without repeated characters (a to c) differ significantly (*p* < 0.05). The data were plotted as the mean ± SD (*n* = 3).

Because oxidative stresses were confirmed (Figure 5, Figure 6 and Figure 7), the source of the oxidative stress needs to be investigated. Cellular antioxidant signaling may regulate oxidative stress [49]. Accordingly, the gene expressions of antioxidant genes [42,45,46] such as *NFE2L2*, *SOD1*, *TXN*, *GSR*, *CAT*, and *GPX1* were evaluated by qRT-PCR following UVC and/or sinularin treatments in oral cancer cells (Ca9-22 and CAL 27) (Figure 8). In general, most of the tested antioxidant gene expressions in these two cell lines were downregulated by separate treatments (UVC or sinularin alone). All of the test antioxidant gene expressions were downregulated by UVC/sinularin. Moreover, the antioxidant gene expressions were lower in the UVC/sinularin treatment than in the separate treatments.

### 3.8. UVC/Sinularin Combined Treatment of Oral Cancer Cells Highly Induces γH2AX and 8-OHdG Expressions

**Figure 9:**
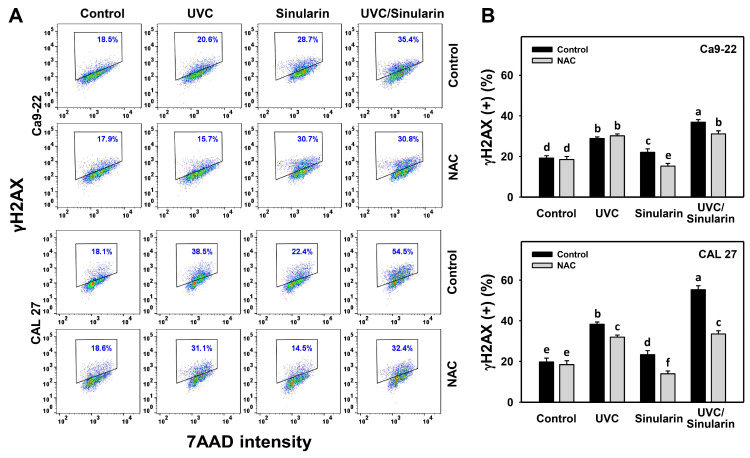
γH2AX expressions of the UVC and/or sinularin treatments. Following the NAC preincubation (5 mM for 1 h) or not, the cells were arranged into four kinds of treatments: the control (0.1% DMSO in medium), UVC, sinularin, and UVC/sinularin for 48 h. The UVC and/or sinularin conditions were 12 J/m^2^, 2 μM and 10 J/m^2^, 3 μM for oral cancer cells (Ca9-22, CAL 27) for 48 h, respectively. (**A**,**B**) Patterns and quantifications for the γH2AX analysis. The trapezoid indicates γH2AX-positive (%). For the multi-comparisons, the treatments marked without repeated characters (a to f) differ significantly (*p* < 0.05). The data were plotted as the mean ± SD (*n* = 3).

**Figure 10:**
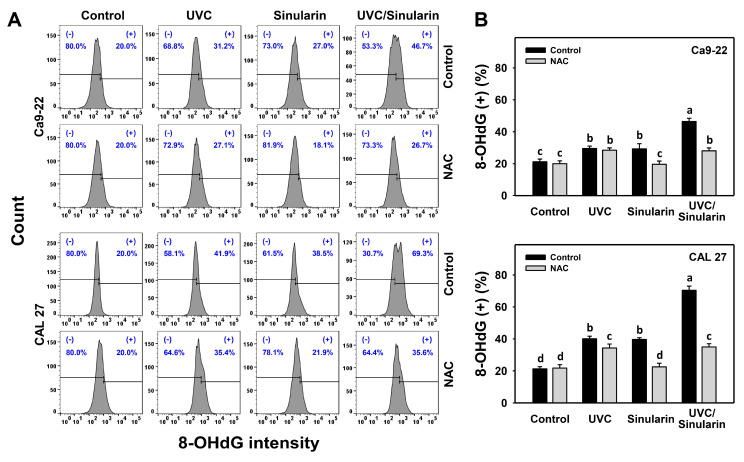
8-OHdG expressions of the UVC and/or sinularin treatments. Following the NAC preincubation (5 mM for 1 h) or not, the cells were arranged into four kinds of treatments: the control (0.1% DMSO in medium), UVC, sinularin, and UVC/sinularin for 48 h. The UVC and/or sinularin conditions were 12 J/m^2^, 2 μM and 10 J/m^2^, 3 μM for oral cancer cells (Ca9-22, CAL 27) for 48 h, respectively. (**A**,**B**) 8-OHdG patterns and quantifications analysis. (+) indicates 8-OHdG-positive (%). For the multi-comparisons, the treatments marked without repeated characters (a to d) differ significantly (*p* < 0.0001). The data were plotted as the mean ± SD (*n* = 3).

In the γH2AX assay (Figure 9A), UVC/sinularin exhibited higher γH2AX (+) (%) in oral cancer Ca9-22 and CAL 27 cells than UVC, sinularin, and the control (Figure 9B). In the 8-OHdG assay (Figure 10A), UVC/sinularin exhibited higher 8-OHdG (+) (%) in oral cancer cells than the other treatments (Figure 10B). Moreover, the involvement of oxidative stress-modulating effect on the γH2AX and 8-OHdG changes was estimated by the presence of NAC (Figure 9 and Figure 10). NAC reverted the γH2AX and 8-OHdG DNA damages of sinularin and UVC/sinularin treatments in both oral cancer cell lines.

## 4. Discussion

Sinularin exhibits the selective killing of cancer cells, and it shows low cytotoxicity to several non-malignant cells [24,25,26], which may demonstrate that sinularin has a low side effect potential for normal cells. A combined treatment with natural products may alleviate radio- and chemo-resistance or side effects in cancer therapy [5,6]. Recently, several potential UVC sensitizers for the antiproliferation of oral cancer cells were reported [13,15,16,17]. Accordingly, the current study evaluated the antiproliferation effect of the UVC/sinularin combined treatment of oral cancer cells. For the MTS and ATP assays, the UVC/sinularin treatment of two cell lines for oral cancer showed high antiproliferation, but normal oral cells were only weakly affected. The detailed mechanism of the synergistic antiproliferation of UVC/sinularin is discussed as follows.

### 4.1. UVC/Sinularin Combined Treatment Synergistically and Selectively Kills Oral Cancer Cells

Several studies reported that several clinical drugs and natural products enhanced the antiproliferation of cancer cells following UVC irradiation. For example, clinical agents such as cisplatin treatment (10 μM) and UVC irradiation (10 J/m^2^) show synergistic suppression for proliferation to colorectal cancer cells [13]. Similarly, several kinds of combined treatments involving UVC and anticancer agents have been reported to provide the synergistic suppression of the proliferation of oral cancer cells, such as methanol extract of *Cryptocarya concinna*/UVC [16], ethyl acetate *Nepenthes* extract/UVC [17], and sulfonyl chromen-4-ones/UVC [15]. Therefore, the drug discovery of UVC radiosensitizers has the potential to improve oral cancer therapy.

Several synthetic agents and natural products have been developed to sensitize radiation effects upon cancer cells [16,50,51]. An ideal radiosensitizer can enhance the radiotherapy efficiency against tumors and is harmless to normal tissues. It is still necessary to identify effective radiosensitizers in oral cancer therapy.

Sinularin is a potential natural product with selective antiproliferation for oral [24] and breast [25] cancer cells. The drug safety of sinularin was demonstrated to provide high viability to normal breast and oral cells at 24 h of treatment [24,25] and to normal oral cells upon more prolonged exposure (48 h) (Figure 1). Moreover, normal oral cells exhibit high viability following UVC/sinularin combined treatment at 48 h (Figure 1), suggesting that such UVC/sinularin combined treatment is tolerated by normal oral cells. UVC/sinularin showed synergistic effects for antiproliferation against oral cancer cells, with the evidence of both MTS and ATP assays at 48 h. Therefore, oral cancer cells following UVC/sinularin treatment exhibit both synergistic and selective killing effects against cytotoxicity to normal cells.

### 4.2. Oral Cancer Cells Following UVC/Sinularin Combined Treatment Induce Higher Oxidative Stress Than the Separate Treatments

For oral cancer cells, UVC irradiation doses ranging from 12 to 14 J/m^2^ have a potential for ROS production [16,17,35] (Figure 5), and sinularin at 23.5 [24], 2 and 3 μM (Figure 5) also induces ROS production. Following the UVC/sinularin treatment, oral cancer cells generate several oxidative stress inductions, such as ROS production, MitoSOX generation, and MitoMP destruction. In contrast, these oxidative stress inductions of UVC/sinularin are minor in normal oral cells. These findings suggest that oral cancer cells following UVC/sinularin treatment selectively induce higher oxidative stress than normal oral cells.

Antioxidant gene expression and oxidative stress interplay in their relationship [52,53]. For example, resveratrol was reported to activate antioxidant signaling to suppress oxidative stress [54]. In contrast, downregulating antioxidant signaling—such as *NFE2L2* [55,56], *SOD1* [57], and *CAT* [58] genes—may trigger oxidative stress. In the current study, antioxidant gene expressions (*NFE2L2*, *SOD1*, *TXN*, *GSR*, *CAT*, and *GPX1*) in oral cancer Ca9-22 and CAL 27 cells were highly downregulated by UVC/sinularin combined treatments compared to the separate treatments (Figure 8). Therefore, the downregulation of antioxidant signaling contributes to oxidative stress in the UVC/sinularin combined treatment.

### 4.3. UVC/Sinularin Combined Treatment in Oral Cancer Cells Induces Higher DNA Damage and Triggers Apoptosis Compared to the Single Treatment

Excessive oxidative stress can lead to DNA damage [59], and may result in the apoptosis of cancer cells [60]. Because oral cancer cells following UVC/sinularin treatment selectively induce oxidative stress, its inducing abilities for DNA damage and apoptosis are expected. Following the detection of γH2AX and 8-OHdG-detected DNA strand damage and oxidative DNA adduct damages, oral cancer cells following UVC/sinularin treatment showed the higher induction of DNA damage than the UVC or sinularin in separate treatments (Figure 9 and Figure 10).

Moreover, oral cancer cells following UVC/sinularin treatment induce higher apoptosis than UVC or sinularin only. Oral cancer cells following UVC/sinularin trigger more apoptosis than normal oral cells. They are supported by the findings of the increment of the subG1 population and annexin V, and the activation for pan-caspase and Cas 3/7 activities. The Western blotting results further demonstrated that UVC/sinularin overexpresses c-PARP in oral cancer cells compared to UVC or sinularin in separate treatments (Figure 4D). Still, it shows a low expression of c-PARP in normal oral cells. Therefore, oral cancer cells following UVC/sinularin treatment selectively cause apoptosis compared to normal oral cells.

### 4.4. UVC/Sinularin Combined Treatment in Oral Cancer Cells Induces Higher G2/M Arrest Than Separate Treatments

Several drugs have been reported to induce G2/M arrest associated with apoptosis, such as luteolin for colon cancer cells [61], erianin for osteosarcoma cells [62], and diosgenin for breast cancer cells [63]. UVC irradiation to oral cancer Ca9-22 cells at 24 h recovery time induces G2/M arrest [17]. Similarly, 24 h sinularin treatment induces G2/M arrest for liver [23], breast [25], and oral [24] cancer cells. The current study shows that oral cancer cells following UVC/sinularin at 48 h treatment exhibit moderate G2/M arrest and apoptosis compared to their separate applications. This warrants the detailed investigation of the contribution of G2/M arrest in UVC/sinularin-induced synergistic antiproliferation for oral cancer cells in the future.

### 4.5. The Combined Effects of UVC/Sinularin in Oral Cancer Cells Depend on ROS Regulation

The ROS scavenger NAC can revert the accompanied changes caused by combined UVC/sinularin treatment in oral cancer cells, including antiproliferation (MTS and ATP assays), G2/M arrest, apoptosis enhancement (annexin V, pan-caspase, Cas 3/7, and apoptosis signal), oxidative stress appearance (ROS, MitoSOX, and MitoMP), and DNA strand damage (γH2AX and 8-OHdG). These findings suggest that the synergistic antiproliferation mechanisms of UVC/sinularin in two oral cancer cell lines are mediated by oxidative stress.

## 5. Conclusions

Combined treatment is an effective strategy to improve cancer therapy. This study first investigated the enhanced and selective antiproliferation by combined treatment using UVC/sinularin in oral cancer cells. Based on the MTS and ATP assays, oral cancer cells following UVC/sinularin combined treatment show synergistic and selective killing. However, it shows low cytotoxicity acting upon normal oral cells in combined as well as separate treatments. Mechanistically, UVC/sinularin in oral cancer cells show higher ROS production, MitoSOX generation, and MitoMP destruction (indicating oxidative stresses) as well as annexin V, pan-caspase, Cas 3/7, and c-PARP expression (indicating apoptosis) than in normal oral cells. UVC/sinularin also shows higher G2/M arrest and γH2AX/8-OHdG expressions (DNA damages) in oral cancer cells than in single treatments. All of these accompanied changes of UVC/sinularin can be reverted by NAC preincubations, demonstrating that the UVC/sinularin-induced synergistic suppression of the proliferation of oral cancer cells is linked to oxidative stress-associated mechanisms. Therefore, a combined UVC/sinularin treatment for oral cancer cells provides a synergistic and selective antiproliferation function with low toxic side damage acting upon normal oral cells.

## Data Availability

Not applicable.

## Supplementary Materials

The following are available online at https://www.mdpi.com/article/10.3390/cancers13102450/s1, Figure S1: The 1H-NMR spectrum of sinularin, Figure S2: The 13C-NMR spectrum of sinularin, Figure S3: The HPLC profiles of sinularin, Figure S4: Original western blotting figures.
