# Supplementary material for: Oxidative Stress-Dependent Synergistic Antiproliferation, Apoptosis, and DNA Damage of Ultraviolet-C and Coral-Derived Sinularin Combined Treatment for Oral Cancer Cells
Source: Cancers (Basel). 2021 May 18;13(10):2450. doi: 10.3390/cancers13102450 (PMC8158103; doi:10.3390/cancers13102450)
Supplement: Supplementary file 1 [file cancers-13-02450-s001.zip › cancers-1180696-supplementary.pdf]

Supplementary Figure S1. The ^{1}H-NMR spectrum of sinularin.

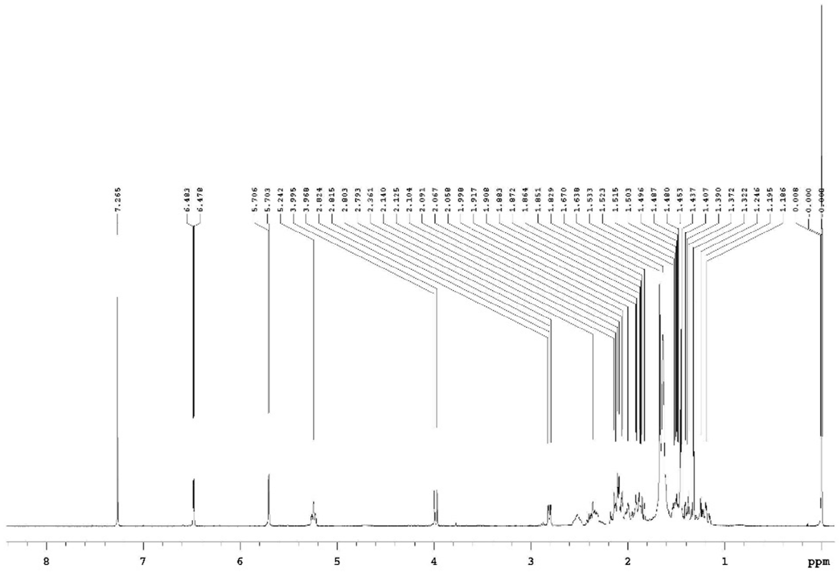

Supplementary Figure S2. The ^{13}C-NMR spectrum of sinularin.

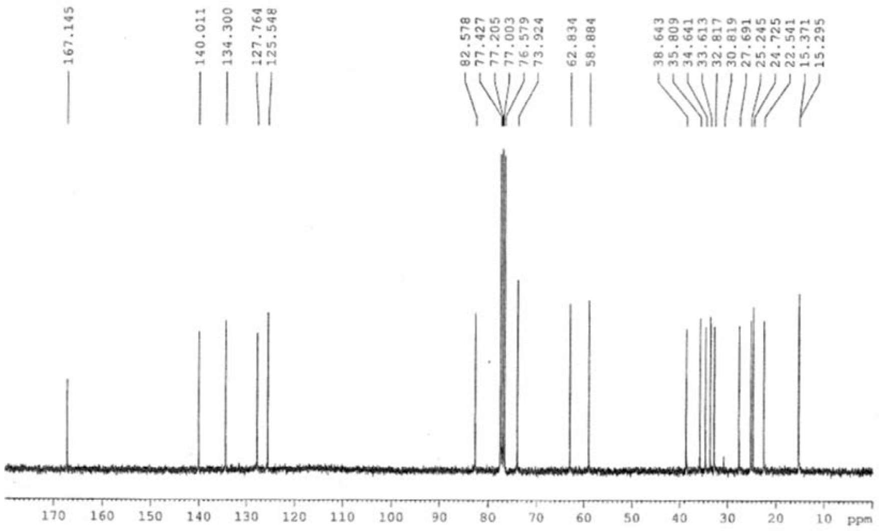

Supplementary Figure S3. The HPLC profiles of sinularin. HPLC had run on a Shimazu HPLC machine with a ChromaNik C-18 column (4.6 mm i.d. x 250 mm in length), the solvent system 75% MeOH aq., and flow rate 1ml/min. Figure S3a is the HPLC profile, and Figure S3b is the purity check.

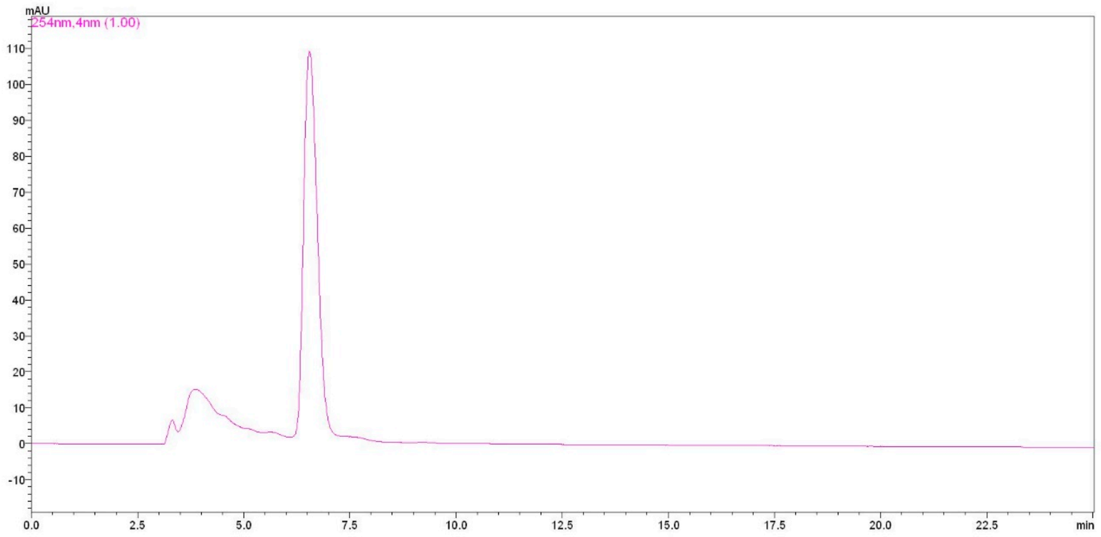

Figure S3a. The HPLC profile of sinularin.

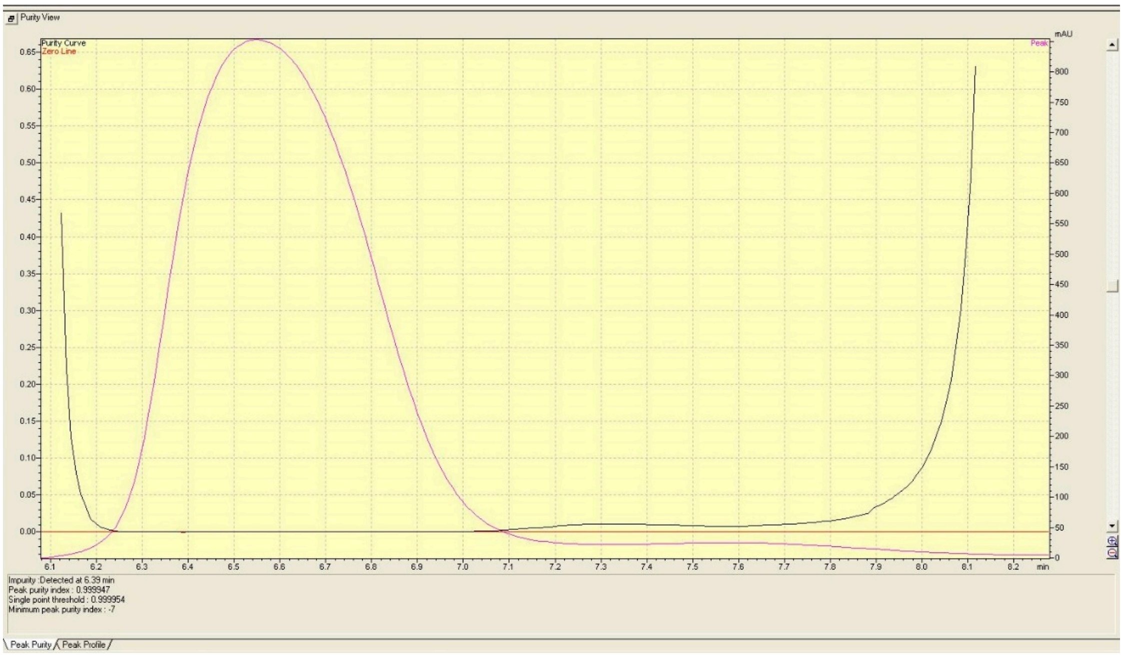

Figure S3b. The calculation on the target peak indicated the purity of sinularin is over 99%. This purity value was shown in the left-bottom.

Supplementary Figure S4. Western blotting raw data for Figures 4D and 4E. (A-C) Whole blot, cut view, and result for Figure 4D. (D-F) Whole blot, cut view, and triplicate data for oral cancer “Ca9-22” cells in Figure 4E. (G-I) Whole blot, cut view, and triplicate data for oral cancer “CAL 27” cells in Figure 4E. (J-L) Whole blot, cut view, and triplicate data for normal oral “HGF-1” cells in Figure 4E.

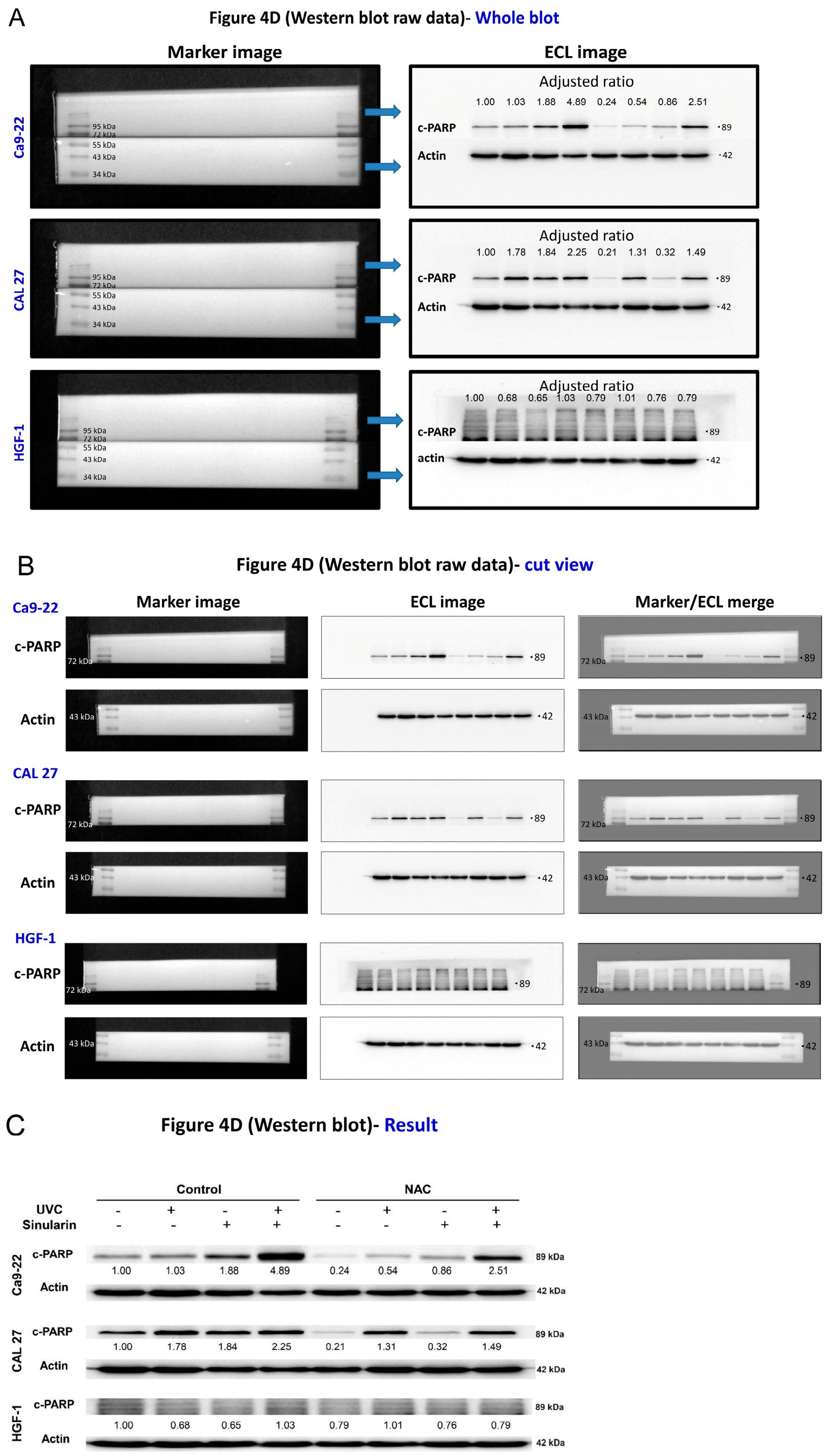

Figure S4(A-C). Whole blot, cut view, and result for Figure 4D.

D

Figure 4E (Ca9-22 Western blot raw data)- Whole blot

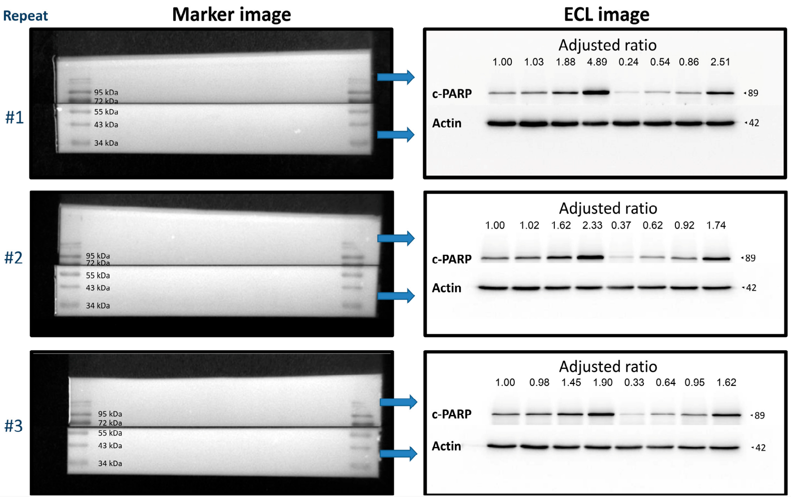

E

Figure 4E (Ca9-22 Western blot raw data)- cut view

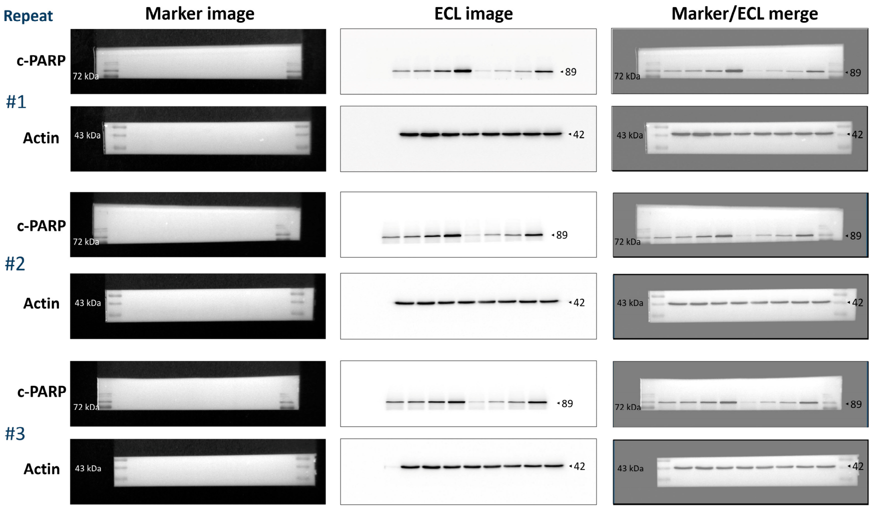

F

Figure 4E Ca9-22 (triplicate data)

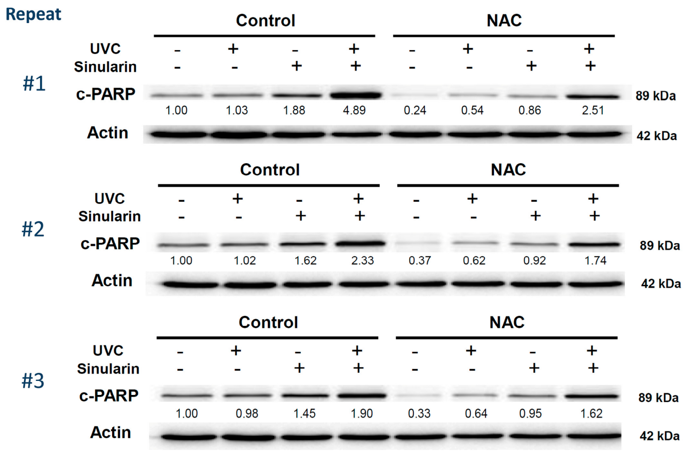

Figure S4(D-F). Whole blot, cut view, and triplicate data for oral cancer “Ca9-22” cells in Figure 4E.

G

Figure 4E (CAL 27 Western blot raw data)- Whole blot

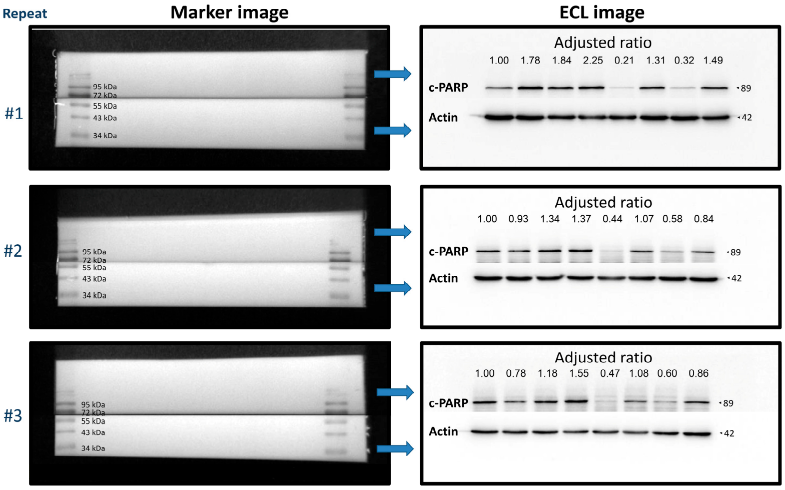

H

Figure 4E (CAL 27 Western blot raw data)- cut view

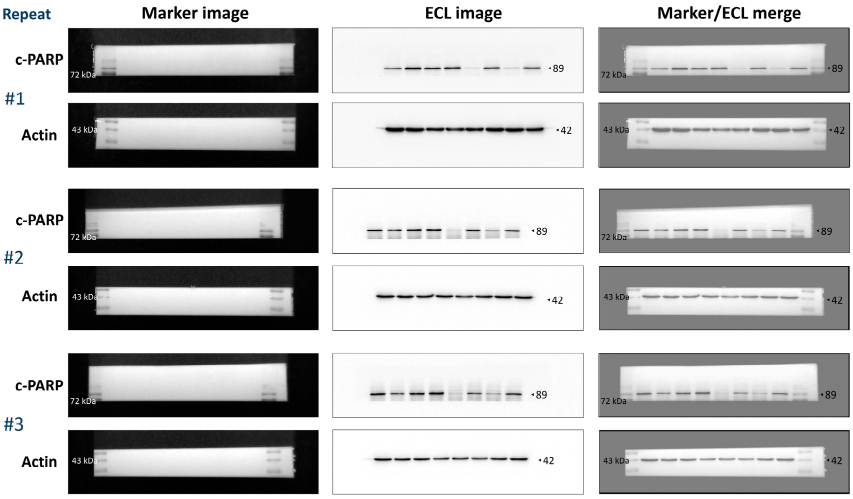

I

Figure 4E CAL 27 (triplicate data)

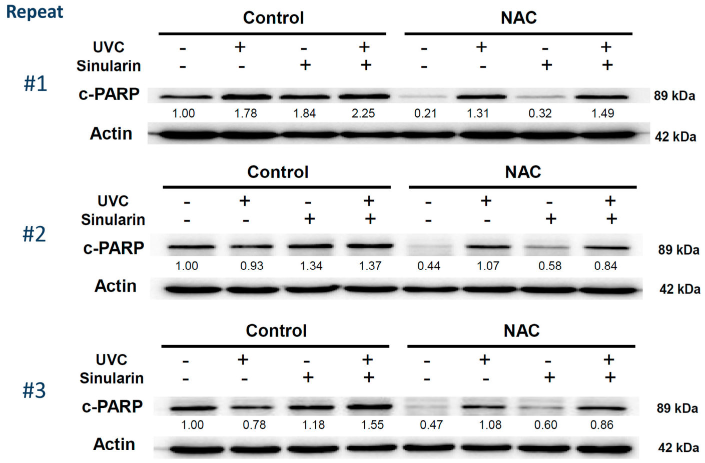

Figure S4(G-I). Whole blot, cut view, and triplicate data for oral cancer “CAL 27” cells in Figure 4E.

J

Figure 4E (HGF-1 Western blot raw data)- Whole blot

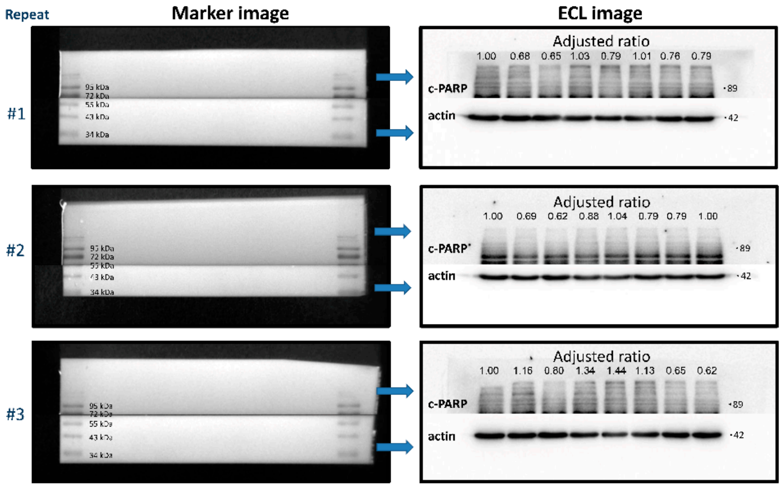

K

Figure 4E (HGF-1 Western blot raw data)- cut view

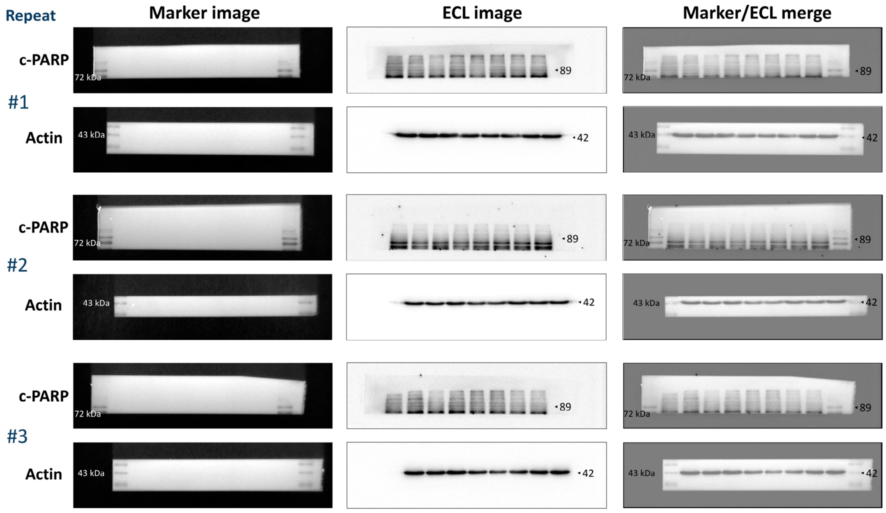

L

Figure 4E HGF-1 (triplicate data)

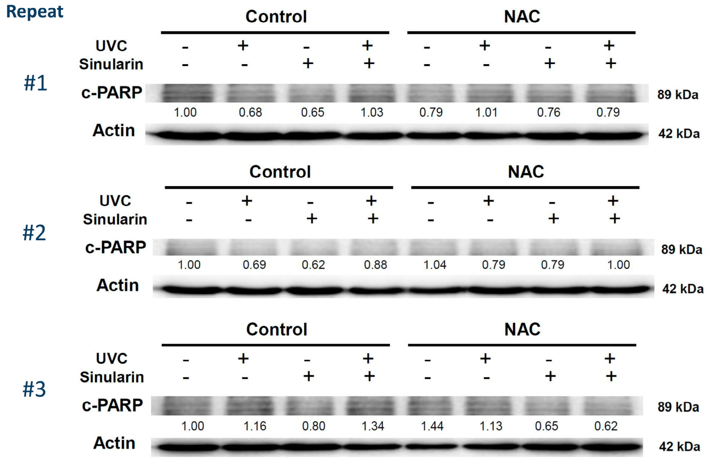

Figure S4(J-L). Whole blot, cut view, and triplicate data for normal oral “HGF-1” cells in Figure 4E.
